# Effects of 28 days of resistance exercise and consuming a commercially available pre-workout supplement, NO-Shotgun^®^, on body composition, muscle strength and mass, markers of satellite cell activation, and clinical safety markers in males

**DOI:** 10.1186/1550-2783-6-16

**Published:** 2009-08-05

**Authors:** Brian Shelmadine, Matt Cooke, Thomas Buford, Geoffrey Hudson, Liz Redd, Brian Leutholtz, Darryn S Willoughby

**Affiliations:** 1 Department of Health, Human Performance, and Recreation, Baylor University, Box 97313, Waco, TX 76798, USA; 2 Institute for Biomedical Science, Baylor University, Waco, TX 87898, USA

## Abstract

**Purpose:**

This study determined the effects of 28 days of heavy resistance exercise combined with the nutritional supplement, NO-Shotgun^®^, on body composition, muscle strength and mass, markers of satellite cell activation, and clinical safety markers.

**Methods:**

Eighteen non-resistance-trained males participated in a resistance training program (3 × 10-RM) 4 times/wk for 28 days while also ingesting 27 g/day of placebo (PL) or NO-Shotgun^® ^(NO) 30 min prior to exercise. Data were analyzed with separate 2 × 2 ANOVA and t-tests (p < 0.05).

**Results:**

Total body mass was increased in both groups (p = 0.001), but without any significant increases in total body water (p = 0.77). No significant changes occurred with fat mass (p = 0.62); however fat-free mass did increase with training (p = 0.001), and NO was significantly greater than PL (p = 0.001). Bench press strength for NO was significantly greater than PL (p = 0.003). Myofibrillar protein increased with training (p = 0.001), with NO being significantly greater than PL (p = 0.019). Serum IGF-1 (p = 0.046) and HGF (p = 0.06) were significantly increased with training and for NO HGF was greater than PL (p = 0.002). Muscle phosphorylated c-met was increased with training for both groups (p = 0.019). Total DNA was increased in both groups (p = 0.006), while NO was significantly greater than PL (p = 0.038). For DNA/protein, PL was decreased and NO was not changed (p = 0.014). All of the myogenic regulatory factors were increased with training; however, NO was shown to be significantly greater than PL for Myo-D (p = 0.008) and MRF-4 (p = 0.022). No significant differences were located for any of the whole blood and serum clinical chemistry markers (p > 0.05).

**Conclusion:**

When combined with heavy resistance training for 28 days, NO-Shotgun^® ^is not associated with any negative side effects, nor does it abnormally impact any of the clinical chemistry markers. Rather, NO-Shotgun^® ^effectively increases muscle strength and mass, myofibrillar protein content, and increases the content of markers indicative of satellite cell activation.

## Introduction

Heavy resistance training in humans enhances muscle protein synthesis [1-3] with concomitant increases in muscle strength and hypertrophy [4-6]. Increases in muscle protein synthesis occurring in response to resistance training can be attributed to pre-translational (increase in mRNA abundance) mechanisms [7], as muscle-specific gene expression is up-regulated in order to provide an ample supply of mRNA template to meet translational (increases in protein synthesis/unit of mRNA) demands. This process is critical since skeletal myocytes are multi-nucleated and each myonucleus controls both mRNA and protein synthesis over a finite sarcoplasmic volume (aka. the myonuclear domain) [8].

Muscle hypertrophy is also regulated by myogenic mechanisms, and in response to resistance training, skeletal muscle hypertrophy can occur through satellite cell activation. During this process, mechanical overload activates satellite cells, which are located between the sarcolemma and basal lamina [9]. These cells then differentiate and proliferate, thereby donating their nuclei to pre-existing myocytes in order to maintain the myonuclear domain [10]. Research in humans indicates that resistance training can increase the number of satellite cells and increase myonuclei in the myofibril [11,12]. As such, resistance training can increase the proportion of satellite cells and the number of myonuclei [12], which suggests that satellite cell activation is an important adaptive mechanism involved in hypertrophy. The activation of satellite cells [13] which accompanies resistance training can be stimulated byvarious signals, including anabolic hormones such as insulin-likegrowth factor I (IGF-I) and hepatocyte growth factor (HGF), regulatory proteins such as the myogenic regulatory factors (MRFs), and nitric oxide. IGF-1 is released from the liver and binds with membrane-bound receptors on the sarcolemma, thereby activating intracellular signaling through the Akt/mTOR pathway. IGF-I has been shown to play a role in myogenesis by stimulating satellite cell proliferation and differentiation [14]. HGF is a heparin-binding growth factor that is localized in the extracellular domain of un-stimulated skeletal muscle fibers, and after stimulation by mechanical overload HGF quickly associates with satellite cells [15]. Furthermore, quiescent and activated satellite cells have been shown to express the c-met receptor, which mediates the intracellular signaling response of HGF. In response to muscle injury, HGF associates with satellite cells and co-localizes with the c-met receptor [15]. Therefore, as HGF becomes available for interaction with the c-met receptor, it up-regulates satellite cell activation.

The MRFs (Myo-D, myogenin, MRF-4, myf5) are a family of muscle-specific transcription factors that play a role in muscle hypertrophy by binding to E-boxes in the promoter region of various sarcomeric genes such as myosin heavy chain, myosin light chain, tropomyosin, troponin-C, and creatine kinase [4] resulting in transactivation of transcription. Furthermore, the MRFs appear to play a role in myogenic activation by inducing myoblast differentiation, as MyoD and Myf5 are believed to be involved in satellite proliferation, and myogenin and MRF-4 are involved in satellite cell differentiation [16]. In contrast to myf5 and Myo-D, myogenin and MRF-4 apparently regulate genes specific to contractile protein [17,18], including genes involved in fast and slow fiber differentiation [19], as myogenin has been found to accumulate in Type I fibers and Myo-D in Type II fibers [20]. Human studies indicate that resistance training increases MyoD, myogenin, and MRF-4 mRNA after acute exercise bouts, and that the expression of MyoD and myogenin are correlated with increases in myofibrillar protein [21]. A study involving 16 wk of resistance training resulted in increased MyoD, myogenin, MRF-4, and myf5 mRNA that were correlated with increased myofiber size [22].

Muscle injury has been shown to increase nitric oxide synthesis which mediates muscle hypertrophy associated with satellite cell activation. Shear forces generated by muscle contraction or retraction of damaged fibers within the basal lamina are thought to stimulate nitric oxide synthase to synthesize nitric oxide, which has been suggested to provide the initial signal for satellite cell activation [15]. As such, this has established a supposed link between mechanical changes in muscle, nitric oxide synthesis, and satellite cell activation.

In addition to improvements in resistance training-related adaptations such as body composition and muscle strength and power, various forms of nutritional supplementation [i.e., creatine, protein, branched-chain amino acids (BCAA), leucine, arginine] are thought to function as either transcriptional co-regulators or as myogenic co-factors, as they have been shown to differentially augment muscle hypertrophy through increases in protein synthesis and/or satellite cell activation. Whey protein and leucine ingested in conjunction with eight wk of resistance training was shown to increase muscle strength beyond that achieved with resistance training and a carbohydrate placebo [23]. Creatine supplemented during 12 wk of heavy resistance training has been shown to augment changes indicative of skeletal muscle hypertrophy, as creatine resulted in increases in MHC Type I, IIa, and IIx protein, respectively, as well as a 58% increase in myofibrillar protein content [24]. Furthermore, creatine was found to significantly increase the expression of myogenin and MRF-4 protein [25]. In a similar study, MRF-4 protein expression was increased after 10 wk of resistance training and creatine supplementation, with the increase in MRF-4 expression being significantly correlated with an increased mean fiber area [26]. After 16 wk of heavy resistance training, creatine supplementation increased satellite cell activation, myonuclear number, mean fiber area, and muscle strength compared to whey protein supplementation and control [27].

Creatine supplementation has been shown to enhance myogenic differentiation by activating the p38 MAPK pathway, which is an intracellular signaling pathway responsible for up-regulating skeletal muscle gene expression in response to muscle contraction. Creatine has also been shown to increase the activity of the Akt/mTOR pathway [28]. The Akt/mTOR pathway is an intracellular pathway involved in increasing muscle protein synthesis. Furthermore, the Akt/mTOR pathway can also be activated by leucine [29]. Consequently, leucine supplementation increased the levels of α-ketoisocaproate (KIC) [30]. KIC blunts the activity of the branched-chain keto-acid dehydrogenase (BCKDH) enzyme complex, which decreases skeletal muscle BCAA oxidation that has been shown to occur during exercise [31]. This is further supported by the fact BCAA have been shown to effectively suppress exercise-induced skeletal muscle proteolysis [32].

Along with the typical resistance training adaptations such as improvements in body composition, and increases in muscle strength and myofibrillar protein content, based on the aforementioned data a nutritional supplement containing creatine, leucine, KIC, and arginine ingested in conjunction with heavy resistance training could conceivably increase muscle hypertrophy through mechanisms associated with increased muscle protein synthesis, decreased muscle proteolysis, and/or satellite cell activation. However, there is a paucity of data demonstrating the effectiveness of such a nutritional product on muscle strength and mass and satellite cell activation. Therefore, the purpose of this study was to determine the effects of 28 days of heavy resistance exercise combined with consuming a commercially available pre-workout supplement, NO-Shotgun^®^, on body composition, muscle strength and mass, myofibrillar protein content, markers of satellite cell activation, and clinical safety markers in males. Based on previous research with individual compounds contained in NO-Shotgun^® ^[15,25-27], we hypothesized that 28 days of heavy resistance training combined with this supplement would preferentially increase muscle strength and mass and stimulate the expression of markers indicative of satellite cell activation, without having any adverse effects on blood clinical chemistry markers.

## Methods

### Participants

Eighteen apparently healthy, recreationally active, non-resistance trained [no consistent (at least thrice weekly) resistance training for one year prior to the study] males with an average age of 22.8 ± 4.67 yr, height of 179.5 ± 6.38 cm, and total body mass of 79.1 ± 16.13 kg completed the study. All participants passed a mandatory medical screening. Participants with contraindications to exercise as outlined by the American College of Sports Medicine and/or who had consumed any nutritional supplements (excluding multi-vitamins) such creatine monohydrate, nitric oxide, hydroxy-beta-methylbutyrate (HMB), various androstenedione derivatives, or pharmacologic agents such as anabolic steroids three months prior to the study were not allowed to participate. All eligible subjects signed a university-approved informed consent document. Additionally, all experimental procedures involved in this study conformed to the ethical considerations of the Helsinki Code.

### Testing sessions

The study included baseline testing at day 0 followed by a follow-up testing session at day 29 in which blood and muscle samples were obtained and where body composition and muscle performance tests were performed.

### Strength assessment

Upper- and lower-body one repetition maximum (1-RM) strength tests were performed using the free weight bench press and angled leg press exercises (Nebula, Versailles, OH), respectively. Initially, an estimated 50% (1-RM) measured from the previous testing 1-RM test, was utilized to complete 5 to 10 repetitions. After a two min rest period, a load of 70% of estimated (1-RM) was utilized to perform 3 to 5 repetitions. Weight was gradually increased until a 1-RM was reached with each following lift, with a two min rest period in between each successful lift. Test-retest reliability of performing these strength assessments on subjects within our laboratory has demonstrated low mean coefficients of variation and high reliability for the bench press (1.9%, intraclass *r *= 0.94) and leg press (0.7%, intraclass *r *= 0.91), respectively.

### Body composition assessment

Total body mass (kg) was determined on a standard dual beam balance scale (Detecto Bridgeview, IL). Percent body fat, fat mass, and fat-free mass were determined using DEXA (Hologic Discovery Series W, Waltham, MA). Quality control calibration procedures were performed on a spine phantom (Hologic X-CALIBER Model DPA/QDR-1 anthropometric spine phantom) and a density step calibration phantom prior to each testing session. The DEXA scans were segmented into regions (right & left arm, right & left leg, and trunk). Each of these segments was analyzed for fat mass, lean mass, and bone mass.

Total body water volume was determined by bioelectric impedance analysis (Xitron Technologies Inc., San Diego, CA) using a low energy, high frequency current (500 micro-amps at a frequency of 50 kHz). Based on previous studies in our laboratory, the accuracy of the DEXA for body composition assessment is ± 2% as assessed by direct comparison with hydrodensitometry and scale weight. Test-retest reliability of performing assessments of total body water on subjects within our laboratory has demonstrated low mean coefficients of variation and high reliability (2.4%, intraclass *r *= 0.91).

### Venous blood sampling and percutaneous muscle biopsies

Venous blood samples were obtained from the antecubital vein into a 10 ml collection tube using a standard vacutainer apparatus. Blood samples were allowed to stand at room temperature for 10 min and then centrifuged. The serum was removed and frozen at -80°C for later analysis.

Percutaneous muscle biopsies (50–70 mg) were obtained from the middle portion of the vastus lateralis muscle of the dominant leg at the midpoint between the patella and the greater trochanter of the femur at a depth between 1 and 2 cm. After sample removal, adipose tissue was trimmed from the muscle specimens, immediately frozen in liquid nitrogen, and stored at -80°C for later analysis.

### Supplementation protocol and dietary monitoring

Participants were assigned to a 28-day supplementation protocol, in double-blind placebo controlled manner. Participants ingested either 27 g/day of placebo (maltodextrose) or 27 g/day of NO-Shotgun^® ^(Vital Pharmaceuticals, Inc., Davie, FL). NO-Shotgun contains a proprietary blend of a number of compounds, but those assumed to target muscle strength and mass are creatine monohydrate, beta-alanine, arginine, KIC, and leucine. For each supplement, the dosage was ingested 30 min prior to each exercise session. For days where no exercise occurs, the full dosage of each supplement was ingested in the morning upon waking. Participants completed supplementation compliance questionnaires and returned empty bottles during the post-study testing session.

For dietary analysis, participants were required to record their dietary intake for four days prior to each of the two testing sessions at day 0 and day 29 blood and muscle samples were obtained. The participants' diets were not standardized and subjects were asked not to change their dietary habits during the course of the study. The four-day dietary recalls will be evaluated with the Food Processor IV Nutrition Software (ESHA, Salem OR) to determine the average daily macronutrient consumption of fat, carbohydrate, and protein in the diet for the duration of the study.

### Resistance-training protocol

Participants completed a periodized 28-day resistance-training program split into two upper-extremity and two lower-extremity exercise sessions each wk for 28 days. This constituted a total of 16 exercise sessions, with eight upper-body and eight lower-body exercise sessions. Prior to each exercise session, participants performed a standardized series of stretching exercises. The participants then performed an upper-extremity resistance-training program consisting of nine exercises (bench press, lat pull, shoulder press, seated rows, shoulder shrugs, chest flies, biceps curl, triceps press down, and abdominal curls) twice per week and a program consisting of seven lower-extremity exercises (leg press, back extension, step ups, leg curls, leg extension, heel raises, and abdominal crunches). Participants performed three sets of 10 repetitions at 70 – 80% 1-RM. Rest periods were two min between exercises and between sets. The resistance exercise sessions were not supervised; however, it was required that each participant completed detailed daily resistance-training logs.

### Whole blood and serum clinical chemistry analyses

Whole blood was collected and immediately analyzed for standard cell blood counts with percentage differentials (hemoglobin, hematocrit, RBC, MCV, MCH, MCHC, RDW, WBC counts, neutrophils, lymphocytes, monocytes, eosinophils, basophils and leukocyte differentials) using a Cell-Dyne 3500 (Abbott Diagnostics, Dallas, TX) automated hematology analyzer. The instrument's flow system was primed and the background counts checked daily to ensure appropriate RBC and WBC linearity. The coefficients of variation for the Cell-Dyne 3500 are 0.8747%, 0.8830%, 0.0296%, 0.7903%, and 0.8534% for neutrophils, lymphocytes, monocytes, eosinophils, and basophils, respectively.

Using a Dade Dimension RXL Analyzer (Dade Behring, Newark, DE), serum samples were assayed for general clinical chemistry markers (total cholesterol, high-density lipoproteins, low-density lipoproteins, triglycerides, albumin, glucose, GGT, LDH, uric acid, BUN, creatinine, BUN/creatinine ratio, calcium, creatine kinase, total protein, total bilirubin, ALP, ALT, and AST). This clinical chemistry analyzer was calibrated daily using liquid assay multiqual (BIO-RAD, Hercules, CA). For all assays mentioned above, the coefficients of variation are less than 5%.

### Serum IGF-1 and HGF analyses

Serum samples were analyzed in duplicate for free/bioactive IGF-1 (Diagnostic Systems Laboratories, Webster, TX) and HGF (Biosource, Camarillo, CA) using an ELISA. For IGF-1, this assay has a sensitivity of 0.06 ng/ml, and does not cross-react with albumins or GH binding proteins. For HGF, the sensitivity is 10 pg/ml. For both IGF-1 and HGF, the subsequent absorbances, which were directly proportional to the concentration of analyte in the sample, were measured at a wavelength of 450 nm using a microplate reader (Wallac Victor 1420, Perkin Elmer, Boston MA). A set of standards of known concentrations for IGF-1 and HGF were utilized to construct standard curves by plotting the net absorbance values of the standards against their respective protein concentrations. By applying a four part parameter curve using MikroWin microplate data reduction software (Microtek Lab Systems, Germany), the free IGF-1 and HGF concentrations in the serum samples were calculated. The overall intra-assay percent coefficient of variation was 4.9% and 3.3% for IGF-1 and HGF, respectively.

### Skeletal muscle phosphorylated c-met content and MRF ELISAs

Approximately 20 mg of each muscle sample was weighed and subsequently homogenized using a commercial cell extraction buffer (Biosource, Camarillo, CA) and a tissue homogenizer. The cell extraction buffer was supplemented with 1 mM phenylmethanesulphonylfluoride (PMSF) and a protease inhibitor cocktail (Sigma Chemical Company, St. Louis, MO) with broad specificity for the inhibition of serine, cysteine, and metallo-proteases.

Muscle homogenate samples were analyzed for phosphorylated c-met (Tyr1230/Tyr1234/Tyr1235) using a phosphoELISA kit (Millipore, Billerica, MA). This sensitivity of this particular assay is reported to be 0.78 U/ml. The absorbances, which are directly proportional to the concentration of c-met in the samples, were measured at 450 nm with a microplate reader (Wallac Victor 1420, Perkin Elmer, Boston MA). A set of standards of known concentrations for c-met were utilized to construct standard curves by plotting the net absorbance values of the standards against their respective protein concentrations. By applying a four part parameter curve using MikroWin microplate data reduction software (Microtek Lab Systems, Germany), the c-met concentrations in the muscle samples were appropriately calculated. The overall intra-assay percent coefficient of variation was 6.89%

The muscle protein expression of the MRFs was assessed through the use of ELISAs. Polyclonal antibodies specific for Myo-D, myogenin, MRF-4, and myf5 (where their target specificities had been verified by Western blotting) were purchased from Santa Cruz Biotech (Santa Cruz, CA). Initially, the antibodies were diluted to 1 μg/ml in coating buffer (Na2CO3, NaHCO3, and ddH2O, pH 9.6) and allowed to incubate at room temperature overnight. Following incubation, the plates were washed (1× phosphate buffered saline, Tween-20), blocked (10× phosphate buffered saline, bovine serum albumin, ddH2O), washed, and then incubated with a secondary antibody (IgG conjugated to HRP) diluted to 1 μg/ml in dilution buffer (10× phosphate buffered saline, Tween-20, bovine serum albumin, ddH2O). After washing, a stabilized TMB chromogen was added and the plates were covered and placed in the dark for the last 30-min prior to being stopped with 0.2 M sulphuric acid. The subsequent absorbances, which are directly proportional to the concentration of the MRFs in the samples, were measured at a wavelength of 450 nm. There were no standards used in these ELISAs, thus no standard curve was created. Therefore, the absorbances relative to muscle weight were assessed and compared as percent changes. The overall intra-assay percent coefficients of variation were 7.12%, 6.47%, 8.03%, and 6.57% for Myo-D, myogenin, MRF-4, and myf5, respectively.

### Myofibrillar protein content

Total cellular RNA was extracted from biopsy samples with a monophasic solution of phenol and guanidine isothiocyanate contained within the TRI-reagent (Sigma Chemical Co., St. Louis, MO), and then isolated with 100% isopropanol. The interphase was removed and total (soluble + insoluble) muscle protein was then isolated from the organic phase with 100% isopropanol and washed with a 0.3 M guanidine HCl/95% ethanol solution. Myofibrillar (soluble) protein was further isolated with repeated incubations in 0.1% SDS at 50°C and separated by centrifugation. Total and myofibrillar protein content were determined spectrophotometrically based on the Bradford method at a wavelength of 595 nm [33]. A standard curve was generated (R = 0.98, p = 0.001) using bovine serum albumin (Bio-Rad, Hercules, CA), and total and myofibrillar protein content was expressed relative to muscle wet-weight [34].

### Total DNA content

Total DNA was isolated from the remaining interphase from the total RNA isolation procedure using 100% ethanol, washed with a 0.1 M sodium citrate/10% ethanol solution, and resuspended in 75% ethanol. The DNA was then solubilized in 8 mM NaOH. The total DNA concentration was determined spectrophotometerically (Helio γ, Thermo Electron, Milford, MA) by optical density (OD) at 260 nm using an OD_260 _equivalent to 50 μg/μl [35]. At a wavelength of 260 nm, the average extinction coefficient for DNA is 0.024 μg/ml; therefore, an OD of 1.0 corresponds to a DNA concentration of 50 μg/ml. The final DNA concentration was expressed relative to muscle wet-weight.

### Reported side effects from supplements

On day 29, participants reported by questionnaire whether they tolerated the supplement, supplementation protocol, as well as report any medical problems and/or symptoms they may have encountered throughout the study.

### Statistical analysis

With the exception of the MRFs, all data were analyzed with separate 2 (group) × 2 (time) univariate ANOVA with repeated measures on the time factor with SPSS for Windows Version 16.0 software (SPSS inc., Chicago, IL). Significant differences among groups were identified by a Tukey HSD post-hoc test. For the MRFs, the percent changes from Day 0 to Day 29 were analyzed with separate independent group t-tests (p < 0.05). A probability level of ≤ 0.05 was adopted throughout.

## Results

### Subject demographics

Twenty participants began the study; however, two dropped out due to reasons unrelated to the study. As a result, 18 participants completed the study. The PL group (n = 9) had an average (± SD) age of 22.77 ± 4.91 yr, height of 179.49 ± 8.32 cm, and total body mass of 79.31 ± 17.35 kg. The NO group (n = 9) had age of 22.88 ± 4.70 yr, height of 179.56 ± 4.33 cm, and total body mass of 78.89 ± 15.87 kg. No significant differences were observed between groups for age (p = 0.46), height (p = 0.32), or total body mass (p = 0.27).

### Dietary analysis, supplement compliance, and reported side effects

The diet logs were used to analyze the average caloric and macronutrient consumption relative to total body mass (Table 1). No significant differences existed between groups for total calories (p = 0.12), protein (p = 0.19), carbohydrate (p = 0.18), or fat calories (p = 0.13); however, significant main effects for Time existed for both groups for total calories (p < 0.001), protein (p < 0.001), carbohydrate (p < 0.001), and fat (p < 0.001).

**Table 1:** Dietary Caloric and Macronutrient Intake

**Group**	**PL Day 0**	**PL Day 29**	**NO Day 0**	**NO Day 29**	**Group**	**Time**	**G × T**
Total Calories (kcal/kg)	33.92 (8.51)	35.67 (8.40)	27.88 (7.47)	28.80 (6.94)	0.13	0.001	0.12

Protein (kcal/kg)	1.39 (0.50)	1.69 (0.47)	1.29 (0.30)	1.56 (0.23)	0.14	0.001	0.19

Fat (kcal/kg)	1.48 (0.47)	1.26 (0.43)	1.09 (0.34)	0.99 (0.29)	0.17	0.001	0.18

Carbohydrate (kcal/kg)	4.81 (1.98)	4.88 (1.43)	3.31 (0.97)	3.85 (1.06)	0.19	0.001	0.13

Data are presented as means and standard deviations of daily caloric values expressed relative to total body mass (kcal/kg). No significant interactions existed for total calories, protein, carbohydrate, or fat calories (p > 0.05). However, significant main effects for time existed for both groups for all four variables (p < 0.001).

All participants appeared to have exhibited 100% compliance with the supplement protocol, and were able to complete the required dosing regimen and testing procedures. Over the course of the 28 days, four participants in PL and four in NO reported side effects. For PL, two participants reported feelings of nausea, one reported a rapid heart rate, and one reported shortness of breath. For NO, two participants reported dizziness, two reported feelings of nausea, two reported headache, two reported a rapid heart rate, one reported shortness of breath, and two reported nervousness.

### Body composition

For total body mass, both groups increased with training (p = 0.001) with a strong trend for NO to be significantly greater than PL (p = 0.062). No training (p = 0.77) or supplement related (p = 0.35) changes were seen with total body water. In addition, no training (p = 0.62) or supplement related (p = 0.23) changes were seen with fat mass; however fat-free mass did increase with training (p < 0.001) and the increases seen with NO were significantly greater than PL (p < 0.001) (Table 2).

**Table 2:** Means, standard deviations, and percent changes for body composition and muscle strength variables in the study.

**Variable**	**PL Day 0**	**PL Day 29**	**% Change**	**NO Day 0**	**NO Day 29**	**% Change**	**Time**	**Group × Time**
Body Weight (kg)	79.31	80.4	1.37	78.57	80.48	2.59	p = 0.001	p = 0.062

	17.35	17.57	0.91	15.84	15.54	1.65		

								

Fat Mass (kg)	14.93	15.07	4.85	15.89	15.52	-1.21	p = 0.62	p = 0.23

	11.84	11.16	8.17	10.92	10.13	6.15		

								

Fat-Free Mass (kg)	54.89	55.84	1.69	53.95	56.46	4.75	p = 0.001	p = 0.001

	6.43	6.79	1.62	6.41	6.23	1.49		

								

Total Body Water (L)	42.82	43.34	1.17	40.61	41.92	3.36	p = 0.77	p = 0.35

	5.73	5.96	2.18	4.55	4.32	3.06		

								

Bench Press (kg/kg)	0.908	0.918	0.73	0.779	0.84	8.82	p = 0.005	p = 0.003

	0.223	0.239	6.92	0.215	0.198	5.34		

								

Leg Press (kg/kg)	3.77	4.21	11.99	3.56	4.22	18.4	p = 0.001	p = 0.10

	0.69	0.73	8.36	0.93	1.14	5.74		

### Muscle strength

Bench press (p = 0.005) and leg press (p < 0.001) strength were both increased with training. For bench press strength, NO was significantly greater than PL (p = 0.003.

### Serum markers of satellite cell activation (IGF-1 and HGF)

Serum IGF-1 was significantly increased with training (p = 0.046); however, NO and PL did not differ relative to IGF-1 (p = 0.86). Serum HGF was also significantly increased with training for NO (p = 0.006), with this increase being significantly greater than PL (p = 0.02) (Table 3).

**Table 3:** Serum and selected muscle variables for the Placebo and NO-Shotgun Groups at Days 0 and 29.

	**PL Day 0**	**PL Day 29**	**% Change**	**NO Day 0**	**NO Day 29**	**% Change**	**Time**	**Group × Time**
Serum IGF-1 (ng/ml)	238.61	246.98	8.58	239.04	259.81	9.34	p = 0.046	p = 0.86

	108.68	122.63	37.3	87.57	97.32	20.01		

								

Serum HGF (pg/ml)	238.54	199.54	-8.71	251.21	344.34	47.42	p = 0.006	p = 0.02

	89.72	75.02	34.06	69.87	232.14	62.49		

								

Muscle c-met (ng/mg)	13.34	14.06	8.55	7.82	12.9	118.55	p = 0.019	p = 0.067

	8.19	9.76	48.34	8.14	9.64	102.49		

								

Myofibrillar Protein (μg/mg)	86.18	108.41	26.34	81.47	135.83	70.39	p = 0.001	p = 0.014

	10.27	26.92	15.06	12.14	18.15	37.66		

								

Total DNA (ug/mg)	27.79	29.59	4.67	27.89	52.37	88.75	p = 0.011	p = 0.041

	5.96	11.35	26.41	3.29	7.74	26.81		

								

DNA/Protein	0.32	0.28	-8.77	0.34	0.39	14.22	p = 0.061	p = 0.14

	0.06	0.12	42.24	0.04	0.09	23.76		

Data are presented as means, standard deviations, and percent changes.

### Skeletal muscle markers of satellite cell activation

**Figure 1:**
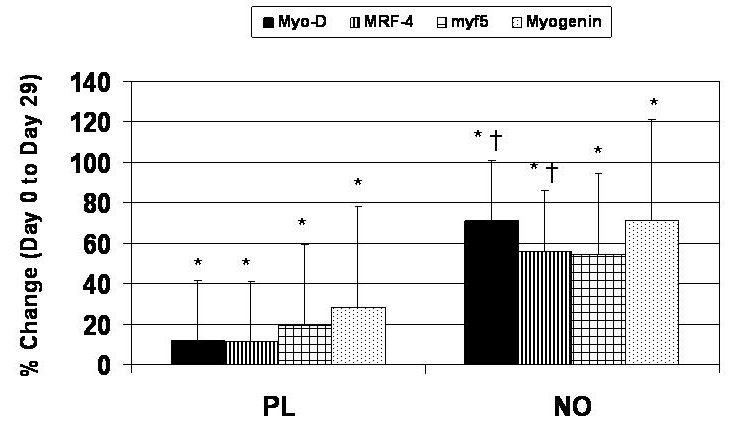
**Data presented as means and standard deviations and expressed as a percent change in absorbance units from Day 0 to Day 29**. All of the MRFs were significantly increased with resistance training in both groups (p < 0.05); however, NO was shown to be significantly greater than placebo for Myo-D (p = 0.008) and MRF-4 (p = 0.022), with weak trends for NO to be significantly greater than PL for myf5 (p = 0.091) and myogenin (p = 0.11). * indicates a significant increase with resistance training. † indicates NO to be significantly greater than PL.

Muscle phosphorylated c-met was increased with training (p = 0.019) with a strong trend for NO to be significantly greater than PL (p = 0.067). For total DNA, both groups increased with training (p = 0.008) and the increases observed in NO were significantly greater than PL (p = 0.042). All of the myogenic regulatory factors were increased with training; however, NO was shown to be significantly greater than PL for Myo-D (p = 0.008) and MRF-4 (p = 0.022 (Figure 1).

### Myofibrillar protein, total DNA content, and DNA/protein

For myofibrillar protein, both groups increased with training (p < 0.001) and the increases observed in NO were significantly greater than PL (p = 0.014) (Table 3). In addition, for total DNA content, both groups increased with training (p < 0.011) and the increases observed in NO were significantly greater than PL (p = 0.041) (Table 3). For DNA/protein, a strong trend was observed but there were no significant changes with training (p = 0.061) and no significant differences between groups (p = 0.14) (Table 3).

### Serum and whole blood clinical chemistry markers

The whole blood and serum markers assessed remained within normal clinical ranges throughout the duration of the study. As a result, no significant differences between groups (p > 0.05) or main effects for Time (p > 0.05) were observed for any of the serum (Table 4) and whole blood (Table 5) clinical chemistry markers.

**Table 4:** Serum Clinical Chemistry Markers for the Placebo and NO-Shotgun Groups at Days 0 and 29.

**Variable**	**PL Day 0**	**PL Day 29**	**NO Day 0**	**NO Day 29**
Triglycerides (mg/dl)	80.63 (37.68)	75.38 (21.67)	108.38 (63.21)	92.25 (46.02)

Cholesterol (mg/dl)	152.25 (23.30)	158.23 (24.27)	179.38 (28.59)	176.63 (25.49)

HDL (mg/dl)	48.13 (8.64)	52.75 (8.82)	53.0 (6.57)	51.88 (8.17)

LDL (mg/dl)	89.38 (18.04)	91.13 (18.58)	106.38 (24.09)	106.5 (21.15)

GTT (U/L)	25.5 (10.07)	25.5 (10.28)	38.0 (36.07)	38.75 (33.70)

LDH (U/L)	109.13 (13.90)	126.0 (41.04)	106.75 (16.56)	112.63 (19.10)

Uric Acid (mg/dL)	5.8 (1.12)	5.5 (1.01)	5.56 (1.02)	5.69 (0.61)

Glucose (mg/dL)	88.38 (6.14)	89.25 (4.59)	90.88 (5.84)	89.13 (5.44)

BUN (mg/dL)	11.88 (3.14)	11.13 (2.30)	14.0 (3.02)	13.13 (3.87)

Creatinine (mg/dL)	0.9 (0.05)	1.04 (0.14)	1.03 (0.10)	1.04 (0.05)

BUN/Creatinine	13.24 (3.61)	10.75 (1.78)	13.75 (2.97)	12.54 (3.31)

Calcium (mg/dL)	8.91 (0.18)	9.03 (0.17)	9.14 (0.20)	9.01 (0.21)

Total Protein (g/dl)	7.31 (0.49)	7.46 (0.37)	7.66 (0.29)	7.59 (0.30)

Total Bilirubin (mg/dl)	0.6 (0.24)	0.53 (0.20)	0.56 (0.36)	0.54 (0.27)

ALP (U/L)	74.88 (25.49)	87.88 (32.30)	61.38 (19.09)	60.88 (18.43)

AST (U/L)	25.88 (20.64)	18.75 (7.19)	15.88 (7.38)	20.25 (14.65)

ALT (U/L)	32.25 (10.70)	29.5 (3.89)	25.88 (3.48)	31.0 (5.76)

CK (U/L)	144.63 (124.81)	138.88 (81.06)	88.88 (47.08)	83.0 (38.15)

Data are presented as means and standard deviations. No significant differences were observed with resistance training or between groups throughout the 28-day study for serum clinical chemistry variables (p > 0.05).

**Table 5:** Whole Blood Clinical Chemistry Markers for the Placebo and NO-Shotgun Groups at Days 0 and 29.

Variable	PL Day 0	PL Day 29	NO Day 0	NO Day 29
WBC (K/μl)	6.10 (1.28)	5.81 (1.35)	5.09 (1.25)	5.37 (2.06)

RBC (M/μL)	5.09 (0.29)	5.27 (0.42)	4.74 (0.26)	4.68 (0.23)

Hemoglobin (g/gl)	15.35 (1.04)	16.08 (1.36)	14.60 (0.68)	14.46 (0.53)

Hematocrit (%)	46.36 (1.92)	48.17 (3.29)	43.41 (1.70)	42.91(1.26)

MCV (fl)	91.11 (1.90)	91.46 (1.81)	91.67 (3.00)	91.90 (4.39)

MCH (pg)	30.13 (1.00)	30.50 (0.81)	30.80 (1.29)	30.91 (1.56)

MCHC (g/dl)	33.10 (1.15)	33.37 (1.03)	33.61 (0.59)	33.62 (0.29)

Lymphocytes (K/μl)	2.07 (0.26)	1.86 (0.43)	1.89 (0.44)	1.54 (0.34)

Monocytes (K/μl)	0.46 (0.15)	0.45 (0.21)	0.27 (0.21)	0.48 (0.24)

Neutrophils (K/μl)	3.34 (1.11)	3.19 (1.15)	2.67 (0.90)	3.02 (2.10)

Eosinophils (K/μl)	0.22 (0.18)	0.23 (0.17)	0.15 (0.11)	0.24 (0.14)

Basophils (K/μl)	0.06 (0.05)	0.06 (0.02)	0.07 (0.04)	0.07 (0.04)

Data are presented as means and standard deviations. No significant differences were observed with resistance training or between groups throughout the 28-day study for whole blood clinical chemistry variables (p > 0.05).

## Discussion

The results of the present study support our hypothesis, indicating that NO-Shotgun^® ^supplementation in conjunction with a 28 days of heavy resistance training, is effective at increasing fat-free mass, muscle strength and mass, myofibrillar protein content, and markers of satellite cell activation, while having no effect on whole blood and serum clinical safety markers in untrained males. Our results agree with previously reported studies that resistance training, when performed in conjunction with creatine [24,25], whey protein and leucine [36], and HMB [37,38] is effective at improving body composition, muscle strength and mass and markers of satellite cell activation.

We observed both NO and PL to significantly increase total body mass (P = 0.001). Additionally, fat-free mass was increased in both groups, and the 4.75% increase in NO was significantly greater than the 1.69% increase in PL. These findings are similar to results observed after 12 wk of heavy resistance training and creatine supplementation, where fat-free mass was increased 9.44% in the creatine group and 1.84% in the carbohydrate placebo group [24]. In addition, 10 wk of heavy resistance training and whey protein and amino acid supplementation resulted in increases in fat-free mass of 5.62% compared to increases of 2.70% for carbohydrate placebo [34].

Relative to muscle strength, we observed NO to increase in bench press and leg press strength by 8.82% and 18.40%, respectively, compared to the respective increases in bench press and leg press strength of 0.74% and 10.30% for PL. However, only bench press was significantly greater for NO compared to PL (p = 0.003). Our observed increases in muscle strength are supported by previous studies which demonstrated heavy resistance training, when combined with creatine [24,27], protein and amino acids (34), and whey protein and leucine [24] to improve strength levels when compared to placebo. However, it should be noted that NO-Shotgun^® ^contains beta-alanine, which has been shown to possibly potentiate the effects of creatine. A recent study has shown that 10 wk of resistance training combined with creatine and beta-alanine produced superior gains in strength and fat-free mass compared to creatine or placebo [39].

In the present study, we also showed that after 28 days of heavy resistance training and supplementation NO underwent increases in myofibrillar protein of 70.39% that were significantly greater than the 26.34% increase in PL (p < 0.001), and that the increases for NO were significantly different than PL (p = 0.014). This is a similar pattern of response from longer-term studies where creatine supplementation, in conjunction with 12 wk of resistance training, resulted in a 57.92% increase in myofibrillar protein content when compared to a maltodextrose placebo group, which only increased 11.62% [24]. In addition, 10 wk of heavy resistance training combined with a protein and amino acid supplement resulted in a 25.03% increase in myofibrillar protein compared to 10.54% for a carbohydrate placebo [34].

We have demonstrated 28 days of heavy resistance training to increase serum IGF-1 by 9.34% and 8.58%, respectively for NO and PL; however, there was no difference between groups. Treating C_2_C_12 _myoblasts with creatine has been shown to increase the expression of the IGF-1 peptide [40]. A positive relationship has been reported between IGF-1 peptide and total DNA content in muscle during resistance exercise due to satellite cell proliferation stimulated by the locally produced IGF-1 [7]. However, while the IGF-I peptide expressed in skeletal muscleincreases muscular protein synthesis and stimulates differentiation of proliferating satellite cells [14,41], it is unclear whether increases in hepatically-derived circulating IGF-1 has any direct effect on muscle hypertrophy. We have previously shown that 10 wk of heavy resistance training combined with a daily supplement containing whey/casein protein and free amino acids increased circulating IGF-1 levels, while also increasing muscle strength and mass [34]. Additionally, 16 wk of resistance training has been shown to increase circulating IGF-1 levels [42]. However, 12 wk of heavy resistance training has been shown to increase muscle strength and mass without any corresponding increases in circulating IGF-1 [43]. Increases in muscle hypertrophy independent of increases in circulating IGF-1 can possibly be explained by a recent study using a liver IGF-1 deficient mouse model, which involves a reduction in serum IGF-1 of approximately 80% [44]. After 16 wk of resistance training, the IGF-1-deficient mice and control mice exhibited equivalent gains in muscle strength, suggesting that performance and recovery in response to resistance training is normal even when there is a severe deficiency in circulating IGF-1.

HGF is a growth factor bound to an extracellular matrix in skeletal muscle [45] that is capable of activating quiescent satellite cells [46]. Serum HGF levels have been shown to increase 24 hr following a single bout of eccentric exercise [47]. In cultured satellite cells, mechanical stretch has been shown to induce the activity of nitric oxide synthase and increase nitric oxide production, which was associated with increases in HGF [48]. In the present study, for serum HGF we observed PL to decrease 8.71% with training, whereas NO increased 47.42%. Based on the fact that NO-Shotgun^® ^contains arginine, an alleged mediator of nitric oxide synthesis, our results may be partially explained on the premise that nitric oxide mediates the release of HGF, and that nitric oxide synthase activity is increased with satellite cell activation.

Skeletal muscle markers of satellite cell activation examined in this study were phospoyrlated c-met (the proto-oncogene receptor for HGF), total DNA, and the MRFs (MyoD, Myf5, MRF-4, and myogenin). While circulating levels of HGF were increased for NO, skeletal muscle phosphorylated c-met was also increased for NO from resistance training by 118.55% (p = 0.019), with a strong trend for NO to be significantly greater than PL (p = 0.067). Increases in the phosphorylation of the HGF receptor, c-met, may be indicative of a possible increase in satellite cell activation. Since HGF levels increased significantly for NO, an increase in the c-met receptor would likely allow for increased binding of HGF.

Resistance training can increase the number of satellite cells and increase myonuclei in the myofiber [11,12]. However, it has been shown that 16 wk of heavy resistance training combined with creatine supplementation augments satellite cell activation, as evidenced by increases in skeletal muscle mean fiber and area myonuclear number to a much greater extent to whey protein or resistance training alone [28]. Furthermore, the creatine group was shown to have the greatest increase in maximal isometric quadriceps contraction strength. Relative to results for the whey protein group, it was shown to undergo greater increases in skeletal muscle mean fiber area and myonuclear number and isokinetic quadriceps strength when compared to the control group.

In the present study, we did not directly assess satellite cell or myonuclear number. Rather, we assessed markers that are considered to be valid indicators of increased satellite cell activation. In so doing, both groups underwent increases in all MRFs with heavy training. However, Myo-D and MRF-4 showed significantly greater increases in NO than PL. For NO, Myo-D increased by 70.91%, MRF-4 increased by 56.24%, myf5 increased by 54.38%, and myogenin increased by 71.17%, while PL only increased Myo-D increased by 11.53%, MRF-4 increased by 11.24%, myf5 increased by 19.45%%, and myogenin increased by 28.15%. This is a noteworthy result, as MyoD and Myf5 are believed to be involved in satellite proliferation, and myogenin and MRF-4 are involved in satellite cell differentiation [17]. Therefore, our results suggest that NO may have been undergoing a greater amount of satellite cell proliferation and differentiation, as indicated by elevated levels of MyoD and MRF-4, respectively.

We have demonstrated in the present study that total DNA content for NO was increased 88.75%, whereas PL was only increased 4.67% with training (p = 0.011), and the increases observed in NO were significantly greater than PL (p = 0.041). During muscle hypertrophy, myonuclei increase sequentially [49] as satellite cells proliferate, fuse with muscle fibers and donate their nuclei, and increase myonuclear number [50]. Consequently, increases in myonuclear number and sarcoplasmic volume are proportional and the myocyte myonuclear domain remains constant, thereby resulting in no appreciable change in DNA/protein and subsequent maintenance in the myonuclear domain. Conversely, because an increase in myonuclear number expands the quantity of DNA available for gene expression and subsequent protein synthesis, the additional myonuclei will facilitate skeletal muscle hypertrophy, thereby resulting in a decrease in DNA/protein as more muscle protein is synthesized from fewer myocytes/DNA [51]. Nuclei within mature muscle fibers are mitotically inactive [52]; therefore, an increase in skeletal muscle DNA content is indicative of myogenically-induced satellite cell activation.

We observed the increases in myofibrillar protein and total DNA content to occur in both groups; however, while DNA/protein was decreased in PL, it was maintained in NO. Both groups also underwent increase increases in the MRFs and phosphorylated c-met, but the increases were greater for NO. This scenario is conceivably attributed to increases in satellite cell activation due to the premise that initial muscle fiber hypertrophy can expand the myonuclear domain as existing myonuclei increase their protein synthesis to support moderate increases in sarcoplasmic volume [12]. However, once a certain limit in the myonuclear domain is reached, further myofiber hypertrophy may only occur as a result of satellite cell activation and the subsequent addition of new myonuclei [42]. Based on our results for the markers of myogenesis and the maintenance of the myonuclear domain, the present data suggest that the muscle hypetrophy occurring in response to 28 days of heavy resistance exercise combined with NO-Shotgun^® ^supplementation appears to be more effective at promoting the myogenic activation of satellite cells than resistance exercise combined with a carbohydrate placebo. IGF-I activates phosphatidylinositol-3 kinase (PI3K) resulting in downstream phosphorylation of Akt [30,53]. Creatine supplementation has also been shown to enhance the differentiation of myogenic C_2_C_12 _cells by activating the p38 MAPK pathway, as the activation of p38 and the transcription factor, myocyte enhancer factor 2 (MEF-2) were increased [29]. The p38 MAPK pathway is an important signaling pathway responsible for up-regulating the expression of various sarcomeric genes in response to mechanical overload. The Akt/mTOR pathway is an important pathway involved in up-regulating translational activity en route to increases in muscle protein synthesis. The Akt/mTOR pathway was activated in C_2_C_12 _myoblasts treated with creatine, as Akt, mTOR, and p70S6 kinase activity were elevated [29]. The Akt/mTOR pathway can also be activated by leucine [30].

Supplemental leucine leads to increased levels of α-ketoisocaproate (KIC) [31], which inhibits the activity of the branched-chain keto-acid dehydrogenase (BCKDH) complex, thereby blunting BCAA oxidation [32] and muscle proteolysis [54] during heavy resistance exercise. It has been shown that 14 days of KIC and beta-hydroxy-beta-methylbutyrate (HMB) supplementation reduced signs and symptoms of exercise-induced muscle damage in untrained males following a single bout of eccentrically-biased resistance exercise [55]. Furthermore, BCAA ingested prior to 60 min of cycling exercise at 50% of maximal work capacity has been shown to attenuate exercise-induced skeletal muscle proteolysis [56].

In regard to clinical safety measures, all of the whole blood and serum markers assessed remained within normal clinical ranges throughout the duration of the study. As a result, we observed no significant differences between PL and NO, indicating NO-Shotgun^® ^to have no deleterious effects with regard to the whole blood and serum variables we assessed. The NO-Shotgun^® ^supplement contains a number of different compounds with many of these having little to no clinical safety data available. However, there are safety data available for creatine. Creatine is well-tolerated in most individuals in short-term studies [57]. Nevertheless, idiosyncratic effects may occur when large amounts of an exogenous substance containing an amino group are consumed, with the consequent increased load on the liver and kidneys [58]. Therefore, concerns have been raised regarding the long-term safety of creatine supplementation. To date, however, studies consisting of durations of nine wk to five yr have not found clinically significant deviations from normal values in renal, hepatic, and cardiac safety markers in healthy individuals [58].

NO-Shotgun^® ^is a nutritional supplement that contains a synergistic blend of compounds, such as creatine, leucine, KIC, and arginine which have been shown in previous studies to be effective at increasing muscle strength and mass, myofibrillar protein content, muscle protein synthesis, and satellite cell activation. Based on the results presented herein, it is difficult to conclude whether any one compound or the additive and/or synergistic effects of various compounds contained in NO-Shotgun^® ^were responsible for eliciting the effects we observed to occur. Therefore, we conclude that 28 days resistance training, when supplemented with NO-Shotgun^®^, has no negative effects on the clinical safety markers assessed, while effectively increasing muscle strength and mass, myofibrillar protein content, and stimulating increases in myogenic markers indicative of satellite cell activation.

## Competing interests

This study was supported by an internal research grant from Baylor University and a product (dietary supplement) donation from VPX Pharmaceuticals (Davie, FL.). The study Principal Investigator (D.W.) received remuneration from the study sponsor; VPX. None of the co-investigators (co-authors) received financial remuneration from VPX. All other researchers declare that they have no competing interests and independently collected, analyzed, and interpreted the results from this study.

## Authors' contributions

BS assisted in coordination of the study, data acquisition, in performing the statistical analysis, and drafting the manuscript. TB, GH, LR, BL, and MC participated in the data acquisition. DSW conceived the study, developed the study design, secured the funding for the project, assisted and provided oversight for all data acquisition and statistical analysis, assisted and provided oversight in drafting the manuscript, and served as the faculty mentor for the project. All authors have read and approved the final manuscript.
